# Antibiogram and Detection of Resistant Genes in *Escherichia coli* Isolated From Meat and Meat Products of Kathmandu, Nepal

**DOI:** 10.1155/ijm/8867884

**Published:** 2025-10-03

**Authors:** Sajin Dangol, Anup Basnet, Ratnaa Shakya, Shreejana Maharjan, Sarda Acharya

**Affiliations:** ^1^Department of Microbiology, St. Xavier's College, Tribhuvan University, Maitighar, Kathmandu, Nepal; ^2^Department of Food Technology and Quality Control, National Food and Feed Reference Laboratory, Babarmahal, Kathmandu, Nepal

**Keywords:** antimicrobial resistance, antimicrobial resistant genes, carbapenem, colistin, ESBL, multidrug resistant

## Abstract

Antimicrobial resistance (AMR) is a global public health emergency attributed to the inappropriate use of antimicrobial agents in human health, animal health, and environmental sectors. With low awareness regarding the consequences of antimicrobial use in livestock farming, improper slaughter practices, and unhygienic meat sales, AMR is circulating through the food chain. The study is aimed at unraveling the scenario of AMR and detecting resistant genes in *Escherichia coli* in meat and meat products in the market of Kathmandu. A total of 120 meat samples, *n* = 30 chicken meat, *n* = 30 buffalo meat, and *n* = 60 frozen meat products were sourced by random sampling between January and June 2024. Then, 54 isolates recovered from the meat samples were identified as containing *E. coli* based on conventional PCR analysis targeting the 16S rRNA gene of *E. coli*. Antimicrobial susceptibility tests revealed 57.4% of the isolates were resistant to tetracycline, followed by ampicillin (44.4%), trimethoprim-sulfamethoxazole, and ciprofloxacin (38.8%). Then, 44.4% of the isolates out of 54 were found to be multidrug-resistant. ESBL testing showed that seven *E. coli* isolates were ESBL producers, and upon colistin agar MIC test, two isolates were resistant to colistin. Conventional PCR analysis for the detection of ESBL-encoding genes confirmed that L4B and L17B contained both *bla_CTX_* and *bla_TEM-52_* ESBL-encoding genes. Isolates L11B, L15B, and L8C harbored *bla_CTX_*, while L12B and FZ100 harbored *bla_TEM-52_* ESBL-encoding genes. *E. coli* isolates L9C and FZ102 both harbored *mcr-1* colistin-resistant genes, while *mcr-2* was not observed. Carbapenem-resistant isolates were not recovered in the study. Isolation of multidrug-resistant *E. coli*, as well as the presence of ESBL and colistin-resistant genes, probably signifies misuse of antimicrobial agents in veterinary practices as well as cross-contamination due to poor hygiene practices. These actions pose a threat to the health of consumers and assist in the spread of AMR genes across microbial populations. Thus, regulatory bodies need to regulate the antimicrobial usage for veterinary applications and promote good hygiene practices in food processing industries, along with the dissemination of awareness to stakeholders regarding AMR.

## 1. Introduction

Pandemics, epidemics, and endemics have been a major part of human history, affecting the human population throughout the world. Europe was once ravaged by the bubonic plague, claiming one-third of the population. Similar histories of infectious disease have been recorded in Asia and other parts of the globe [1]. The discovery of antibiotics emerged as a breakthrough that revolutionized disease treatment. This finding empowered humans with a means to tackle infections, which were once fatal [2]. The discovery of penicillin by Sir Alexander Fleming laid the foundation for the golden era of antibiotics and reshaped our understanding of disease treatment [3]. The wave of antibiotic discovery triggered by penicillin contributed to the development of a wide range of antibiotics for therapeutic applications. Misuse of these antimicrobials has led to the development of resistance against antimicrobial agents. Drug-resistant bacteria have drastically lowered the efficacy of therapeutic agents, which has led to the evolution of multidrug-resistant (MDR) bacteria [4].

WHO has classified antimicrobial resistance as a World Public Health Emergency in response to the severity of the threat against humans, animals, and the environment as a whole. Emergence and proliferation of resistant pathogens limit the efficacy of medication as well as compromise the success of diverse lifesaving medical procedures, including organ transplants, surgeries, and others [5]. In 2019 in Nepal, mortality attributable to AMR accounted for 6400 deaths, while mortality associated with AMR accounted for 23,200 deaths. AMR ranked third in terms of deaths after cardiovascular diseases and chronic respiratory diseases in 2019. *Klebsiella pneumoniae*, *Staphylococcus aureus*, *Escherichia coli*, *Pseudomonas aeruginosa*, and *Streptococcus pneumoniae* represent the big five pathogens in Nepal that result in the majority of the mortality associated with AMR parenthesis. Out of total deaths reported from AMR, *E. coli* was the third major causative agent for mortality with 3200 deaths in 2019 [6].

The human population has exceeded 8 billion, which has created strain on the food supply chain. To meet the ever-increasing demand for animal products, the use of antibiotics has skyrocketed in livestock farming [7]. Two-thirds of the global antibiotic consumption is solely dedicated to livestock farming worldwide [8]. The increasing rate of antibiotic application in meat production is one of the contributing factors in the emergence of antimicrobial resistance [9]. Exposure to antibiotics creates a strong driving force and pressure, which creates conditions favorable for the emergence of resistant genes [10]. Tetracyclines account for 28%, aminoglycosides for 23% and fluoroquinolones for 15% of the overall import and sale of antimicrobials for veterinary purposes in Nepal. In the study “Knowledge, Attitude, and Practice of Antibiotic Use and Resistance among Poultry Farmers in Nepal” by Subedi et al., in 2023, 87.8% poultry farmers confirmed that they use antimicrobial agents in poultry, 49.8% of them were aware of AMR while 55.7% knew that haphazard employment and misuse of antimicrobials could have consequences to human health and the environment [11]. Nepal banned the use of antibiotics as a growth promoter in 2017. In 2016, the Ministry of Health and Population approved the National Antibiotic Containment Plan and the National Antibiotic Treatment Guideline in 2014, which were aimed at human health. While the regulation of antimicrobials in veterinary practice is still lagging, the knowledge, attitude, and practice (KAB) study performed in the poultry sector reported 9.2% of farmers never used antimicrobials throughout the production cycle, while 90.8% used antimicrobials at least once.

A major concern in developing countries like Nepal is the purchase and application of drugs directly from drug sellers without a prescription. The KAB study revealed that 50.1% of farmers obtained the antimicrobials without any prescription from drug sellers [12].

*E. coli* is an opportunistic pathogen that is associated with intestinal and extraintestinal diseases. *E. coli* is responsible for diarrhea, enteritis, bacteremia, urinary tract infections, and other infections in humans and animals [13, 14]. Then, 700 and even more strains or serovars of *E. coli* exist in various habitats, including food and water, of which only a few are of major concern to human health. O157:H7, a causative agent of bloody diarrhea, non-O157:O7 strains—O26, O145, O111, O121, and O45, and shiga toxin-producing *E. coli* are of major concern, as they have been frequently reported in various foodborne outbreaks and infections [15].

The occurrence of foodborne outbreaks is common in developing countries, including Nepal. Foodborne diseases are prevalent due to poor hygiene and sanitation practices, inadequate food laws and regulatory mechanisms, lack of awareness among meat handlers, and poor safety equipment. Unhygienic slaughter practices and poor meat processing facilities lead to cross-contamination of meat and meat products. The status of implementation of the WHO-FAO Codex Alimentarius guidelines for safe and hygienic production of meat from the beginning of the production line to retail shops for consumption in Nepal is lacking [16].

Surveillance and investigation of indicator *E. coli* can provide important insight into the resistance pattern that can prevail in other intestinal flora, which may be transferred between animals, the environment, and humans. Results from the study can provide indirect conclusions on resistance determinants like plasmids and genes that can be transferred between large groups of bacterial communities. These bacteria can further infect humans and animals. Thus, investigation of antimicrobial resistance in *E. coli* recovered from meat and meat products, along with detection of susceptibility patterns and resistant genes—extended-spectrum beta-lactamases (ESBLs) and carbapenem-resistant and colistin-resistant genes, can be crucial in understanding the scenario of AMR in meat industries. Resistant genes located in bacterial plasmids can pose a serious threat to the spread of antimicrobial resistance among the bacterial community. Detection of such genes in *E. coli* isolates recovered from meat and meat products can point towards selective stress exerted by the application of antimicrobial agents in food-producing animals or the acquisition of resistance determinants.

## 2. Materials and Methods

A total of 120 meat and meat products collected from meat vendors/shops of Kathmandu, Nepal, were analyzed in the study. Then, 60 raw meat samples and 60 meat products were selected, and analysis of the samples was carried out in the Center for Health and Disease Studies (CHDS) and the Microbiology Laboratory of St. Xavier's College. The study duration was between January 2024 and June 2024.

### 2.1. Study Area

The study was performed in Kathmandu Metropolitan City, a local administrative unit in Kathmandu Valley, along with 15 other municipalities in Bagmati Province of Nepal. As per the article by The Kathmandu Post in 2016, there were around 2500 meat shops and 1000 fish shops in the valley. Exact surveillance of the meat shop has not been done in Nepal; thus, accurate data regarding the meat shops is not available. At present, the number of meat shops can be significantly higher than this number. Live animals are imported from Heatuda, Birgunj, Bara, Chitwan, Nuwakot, Nepalgunj, Dhading, and other cities into the Valley. The study incorporated the meat vendors from different locations within the Kathmandu metropolitan area.

### 2.2. Study Design and Sample Size

The study was a cross-sectional study, and a random sampling plan was used to collect samples between January 2024 and June 2024. Samples were collected from meat vendors selling only one kind of meat. Thus, a total of 120 samples were collected from the vendors around the Kathmandu Metropolitan: chicken meat (*n* = 30), buffalo meat (*n* = 30), and processed meat products (*n* = 60).

### 2.3. Preparation of Sample

Buffered peptone water (BPW) was used as dilution media for the preparation of the initial suspension. Then, 25 grams of the meat sample was weighed and mixed with 225 mL of BPW buffer in a stomacher bag and blended on a stomacher for 1 min on a normal setting [17].

### 2.4. Isolation of *E. coli*

Then, 1 mL of the mixed/macerated test portion was inoculated into 10 mL of single-strength MacConkey Broth medium. Inoculated tubes were incubated at 37°C for 24 h. Following incubation, the tubes were observed for the growth of the organisms with fermentation of lactose in MacConkey Broth. Fermentation was detected by the development of gas in the Durham's Tube. A loopful of the medium was streaked onto Eosin Methylene Blue Agar and incubated overnight at 37°C [17, 18].

### 2.5. Identification of *E. coli*

#### 2.5.1. Colony Morphology

After overnight incubation, the selective agar plate was examined for colony characteristics. The suspected colonies were smooth, small, and had a green metallic sheen on EMB Agar.

#### 2.5.2. Microscopy

Gram staining was performed for the suspected colonies. Biochemical analysis was carried out for isolates observed as Gram-negative rods.

#### 2.5.3. Biochemical Analysis

Biochemical analysis for *E. coli* included tests for motility, indole production, methyl red, Voges–Proskauer reaction, citrate utilization, and TSI agar [17, 18].

### 2.6. Genomic DNA Extraction

Genomic DNA extraction from *E. coli* isolates was carried out by the phenol/chloroform method. Each bacterial isolate recovered (2–3 colonies) was inoculated into LB broth following QC and incubated at 37°C for 16 to 18 h. The culture, after incubation, was centrifuged at 10,000 rpm for 5 min, and then, the supernatant was discarded. The pellet formed was resuspended in 567 *μ*L Tris-EDTA (TE) buffer and mixed. Then, 30 *μ*L of 10% SDS, followed by 3 *μ*L proteinase K at 20 mg/mL, was added, leading to a final concentration of proteinase K of 100 *μ*g/mL in 0.5% SDS. The solution was mixed and incubated at 37°C for 1 h. To the above preparation, 100 *μ*L of 5 M NaCl was pipetted into and mixed vigorously. Addition of 80 *μ*L of CTAB/NaCl solution was performed, thoroughly mixed, and incubated at 65°C for 10 min. An approximately equal volume (0.7–0.8 mL) of chloroform/isoamyl alcohol solution was added, mixed thoroughly, and centrifuged at 12,000 rpm for 5 min. Aqueous, viscous supernatant was pipetted into a fresh Eppendorf tube without disturbing the interface. To this, an equal volume of phenol/chloroform/isoamyl alcohol was added and centrifuged for 5 min at 12,000 rpm. The upper supernatant was transferred into a fresh Eppendorf tube to which 0.6 volumes of isopropanol was added for precipitation of nucleic acid. The tube was mixed thoroughly until a stringy white DNA precipitate was visible. The tube was centrifuged at 12,000 rpm for 5 min, and the supernatant was poured off. To this, 100 *μ*L of 70% ethanol was added, and the mixture was centrifuged at 12,000 rpm for 5 min. The pellet was allowed to air dry following removal of the supernatant fluid. The pellet was redissolved in 100 *μ*L TE buffer and stored at 4°C or −20°C [19].

### 2.7. Molecular Identification of *E. coli*

Molecular identification of *E.* coli isolates was done by PCR amplification of the genomic DNA using specific primers (**16E1-F: 5**⁣′**-GGGAGTAAAGTTAATCCTTTGCTC-3**⁣′** and 16E2-R: 5**⁣′**-TTCCCGAAGGCACATTCT-3**⁣′) targeting a 584-bp fragment of the 16S rRNA gene [20]. *E. coli* strain ATCC 25922 was used as a control. Amplified DNA was separated on a 1.5% agarose gel and visualized under UV light [20]. The PCR conditions for amplification of the 16S rRNA gene of *E. coli* are provided in Table 1.

### 2.8. Antimicrobial Susceptibility Pattern

Antimicrobial susceptibility testing was performed as recommended by the Clinical Laboratory Standard Institute (CLSI) using the Kirby–Bauer agar disk diffusion method. Thirteen (13) different types of antimicrobial agents belonging to different classes were included in the panel of antimicrobials used for the determination of susceptibility patterns. Clinical breakpoints as per the CLSI were used to interpret the inhibition zones (sensitive, intermediate, and resistant) [21].

### 2.9. Test for ESBLs

*E. coli* isolates were screened for ESBL production as per the criteria mentioned in the CLSI guideline 2024. A presumptive ESBL production test required a screening test, which was carried out using cefotaxime (30 *μ*g) or ceftriaxone (30 *μ*g) using the disk diffusion method. The standard disk diffusion procedure was followed, and incubation was at 35°C–37°C for 18–24 h. A zone of inhibition for cefotaxime ≤ 27 mm and ceftriaxone ≤ 25 mm was interpreted as indicating potential ESBL producers. A confirmatory ESBL production test was performed by the combination disk diffusion method, which employed antibiotics—ceftazidime and cefotaxime, alone and in combination with clavulanic acid. The standard disk diffusion procedure was followed with incubation at 35°C–37°C for 16–18 h. An increase in a zone diameter by ≥ 5 mm for either antimicrobial agent tested in combination with clavulanic acid, compared to the zone diameter of the agent tested alone, was confirmed as indicative of ESBL production [21].

### 2.10. Test for Carbapenem Inactivation

According to the protocol established by the CLSI for the modified carbapenem inactivation test, carbapenem resistance was assessed in the recovered *E. coli* isolates. Then, 1 *μ*L loopful of test isolate was emulsified in 2 mL of TSB and vortexed for 15 s. A 10-*μ*g meropenem disk was added to the tube, ensuring that the disk was fully immersed in the suspension. This preparation was then incubated at 35°C ± 2°C for 4 h ± 15 min. Before completion of the incubation period, a 0.5 McFarland suspension of *E. coli* ATCC 25922 was prepared in sterile normal saline. An MHA plate was inoculated with *E. coli* ATCC 25922, carpet culture was performed, and plates were allowed to dry for 3–10 min. The meropenem-containing disk was removed from the TSB-meropenem disk suspension using a sterile loop. The excess liquid was expelled using a loop by dragging and pressing the loop along the inside edge of the tube. The meropenem disk was placed on an MHA plate previously inoculated with *E. coli* ATCC 25922. An MHA plate was then incubated at 35°C ± 2°C for 24 h. A zone diameter of 6–15 mm was interpreted as carbapenemase positive, and a zone diameter of ≥ 19 mm as negative, that is, sensitive to carbapenem, and a zone diameter of 16–18 mm as intermediate [21].

### 2.11. Test for Colistin Resistance

Colistin susceptibility was determined using the agar dilution MIC method, as per the methodological guidelines by the CLSI (2020). Using colistin sulfate, MHA plates with varying concentrations of colistin were prepared. For the test, it required 4 MHA plates with varying concentrations of colistin in them—0 *μ*g/mL (growth control), 1 *μ*g/mL, 2 *μ*g/mL, and 4 *μ*g/mL colistin. A 0.5 McFarland suspension of the test isolate was prepared in sterile normal saline, which was diluted in saline in a 1:10 ratio. A colistin agar plate of each concentration was divided into 10 parts to test up to 10 isolates per plate. Then, 10 *μ*L of the test suspension was streaked onto each colistin agar plate and incubated at 33°C–35°C for 16–20 h. Growth of the isolates in a plate with colistin concentration ≥ 4* μ*g/mL was regarded as resistant, while ≤ 2 *μ*g/mL was considered an intermediate value [21].

### 2.12. Polymerase Chain Reaction Amplification of Colistin Resistance, ESBL, and Carbapenem Resistance Genes

Multiplex PCR was executed separately for the confirmation of the presence of the following:

• Set A: colistin-resistant genes—*mcr-1* and *mcr-2*
• Set B: ESBL-producing genes—*bla_CTX_* group and *bla_TEM-52_*
• Set C: carbapenemase-producing genes—*bla_OXA-48_* and *bla_IMP_*

Table 2 illustrates the final volume of the PCR reaction mixture, the volume of each constituent added to it, and their final concentration.

The Multiplex PCR protocol with PCR conditions [23] is shown in Tables 3 and 4.

### 2.13. Visualization of the PCR Products

Agarose gel electrophoresis was performed for the visualization of the PCR products. An agarose gel 1.5% *w*/*v* was prepared in 1x tris-acetic acid-ethylene diamine tetra-acetic acid (TAE) buffer. The gel was stained with ethidium bromide (0.5 *μ*g/mL), and wells were prepared. Then, 5 *μ*L of amplicons was stained with 1 *μ*L of loading dye and loaded into the agarose wells. Additionally, 5 *μ*L of a 100-bp DNA ladder, 1 *μ*L negative control, and 5 *μ*L positive control were loaded into the wells of the agarose gel. Electrophoresis was conducted at 60 V for 15 min, followed by 90 V for 45 min. The agarose gel was transferred into a UV transilluminator for the visualization of amplicon bands for the confirmation of target genes.

### 2.14. Data Management and Analysis

Results obtained from lab analysis were systematically uploaded into a Microsoft Excel 2019 spreadsheet, and statistical analysis was performed.

## 3. Results

**Figure 1:**
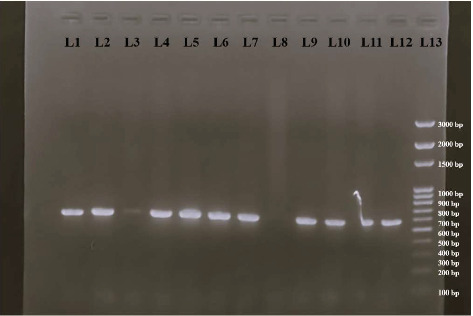
Visualization of PCR products of 16E1–16S rRNA genes of *E. coli* isolates. L1–L11: PCR products of 16S rRNA of *E. coli* isolates; L12: PCR product of 16S rRNA of E. coli ATCC 25922 (Positive control); L13: 100-bp DNA ladder.

In total, 120 meat and meat products were sampled and analyzed for the isolation of *E. coli*. Upon analysis, 93 bacterial isolates were obtained from 120 samples, which were further processed for identification of *E. coli* species. Identification of the bacterial isolates was carried out by selective growth in selective media, followed by microscopic observation for Gram-negative rod-shaped bacteria and biochemical analysis—indole, methyl red, VP, and citrate utilization tests. Out of 93 Gram-negative rods recovered, 58 isolates were identified as *E. coli* based on the results obtained from biochemical tests. Then, 21 isolates were recovered from raw buffalo meat, 20 isolates from raw chicken meat, and 17 isolates from frozen meat products. Then, 54 isolates were confirmed as *E. coli* based on molecular analysis of 16E1 gene, targeting 16S rRNA of *E. coli*, 21 from raw buffalo meat, 17 from raw chicken meat, and 16 isolates from processed frozen meat samples.  Figure 1 shows the confirmation of the isolates as *E. coli*, upon PCR amplification of the 16E1 gene targeting 16S rRNA and visualization under gel doc.

### 3.1. Antimicrobial Susceptibility Pattern of the *E. coli* Isolates

**Figure 2:**
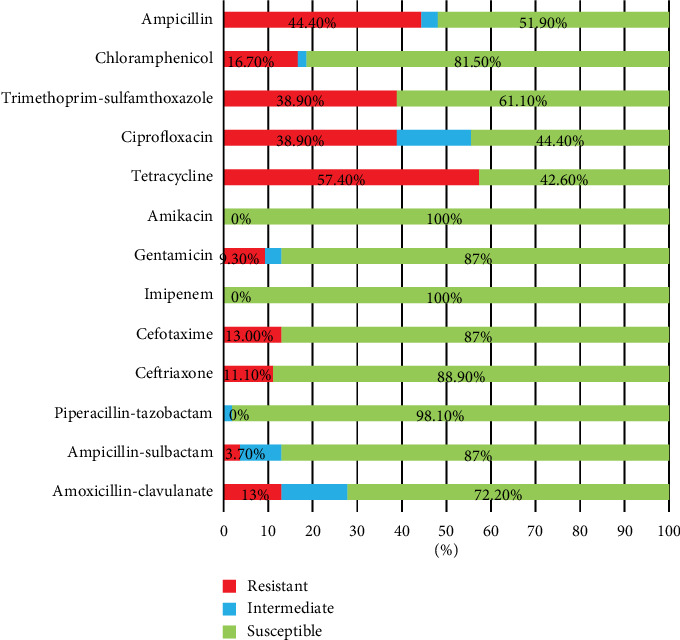
Chart depicting the susceptibility pattern of the *E. coli* isolated from meat samples.

**Figure 3:**
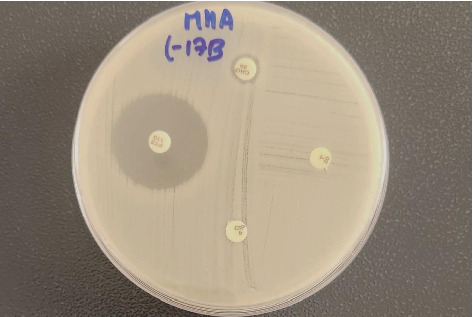
Antimicrobial susceptibility pattern of *E. coli* isolate–L17B. PTZ, piperacillin–tazobactam; CRO, ceftriaxone; CIP, ciprofloxacin; T, tetracycline.

For the determination of antimicrobial susceptibility patterns, *E. coli* isolates were tested against a panel of 13 different antimicrobial agents belonging to nine different antimicrobial classes. Among the tested antimicrobials, amikacin, piperacillin-tazobactam, and imipenem were found to be most effective, where all of the isolates were 100% susceptible. Higher resistance was observed against tetracycline, with 31 isolates (57.4%). Following tetracycline, isolates demonstrated maximum resistance towards ampicillin (24 isolates, 44.4%), ciprofloxacin (21 isolates, 38.8%), and trimethoprim-sulfamethoxazole (21 isolates, 38.8%). The overall susceptibility pattern of the *E. coli* isolates is depicted in Figure 2. The photograph of the antimicrobial susceptibility demonstrated by *E. coli* isolates L-17B is shown in Figure 3.

### 3.2. Comparative Analysis of Antimicrobial Susceptibility Pattern of *E. coli* Based on Meat Source

**Figure 4:**
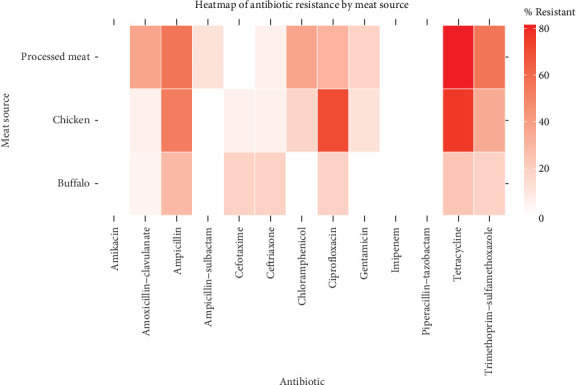
Antimicrobial resistance pattern observed in *E. coli* isolates recovered from different meat sources.

All *E. coli* isolates from three different meat sources were susceptible to imipenem, amikacin, and piperacillin-tazobactam. The resistance pattern of isolates from processed meat products was comparable to that of isolates from raw chicken meat. Variation was observed between the resistance patterns of *E. coli* recovered from processed meat and raw buffalo meat. More resistant *E. coli* were recovered from processed meat compared to other sources. The heat map shown in Figure 4 further illustrates the relationship between the antibiotic susceptibility pattern in *E. coli* recovered from different meat sources.

### 3.3. MDR in *E. coli* Isolates

**Figure 5:**
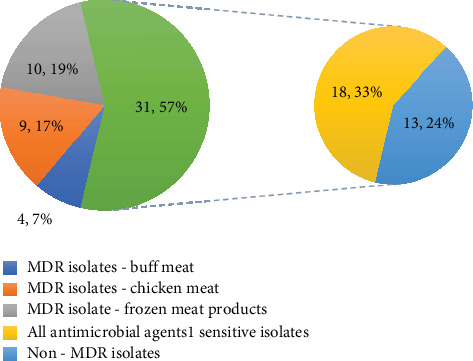
Distribution pattern of the multidrug resistance tendency in *E. coli* isolates in meat sample types.

As illustrated in Figure 5, 44.4% of the *E. coli* isolates recovered from meat samples were identified as MDR isolates, with the highest occurrence of these isolates in frozen meat, especially in frozen chicken meat dumplings. Processed raw chicken cut meat and raw chicken meat categories, where both accounted for 10 MDR *E. coli* isolates—43.4% of MDR isolates and 18.5% of total isolates, respectively. The least MDR was observed in raw buffalo meat, with four *E. coli* isolates being MDR (7%).

### 3.4. Phenotypic ESBL Producers, Colistin Resistance, and Carbapenem Inactivation

**Figure 6:**
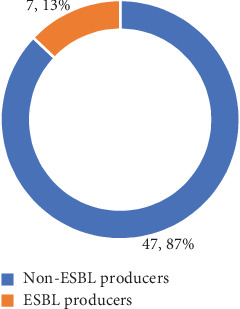
Chart showing total *E. coli* isolates confirmed as ESBL producers.

**Figure 7:**
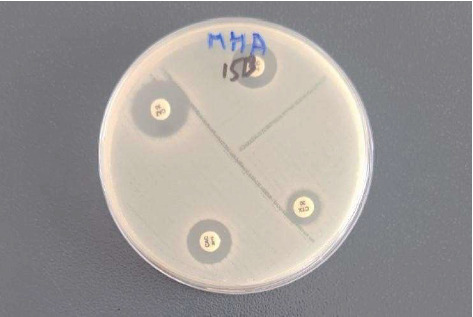
Extended spectrum beta-lactamase production test of E. coli isolate–L15B.

Out of 54 *E.* c*oli* isolates, seven isolates were identified as probable ESBL producers. Upon confirmatory ESBL production testing, all 7 isolates (13%)—L4B, L11B, L12B, L15B, L17B, L8C, and FZ100 were confirmed as ESBL producers, which is demonstrated in Figure 6, and a photograph of the ESBL test is shown in Figure 7. None of the *E. coli* isolates recovered from meat samples were observed to be resistant to carbapenem. In the case of colistin, resistance was shown by two isolates—L8C and FZ102, while the rest of the isolates were susceptible to colistin.

### 3.5. Detection of Resistance-Encoding Genes in *E. coli* Isolates

**Figure 8:**
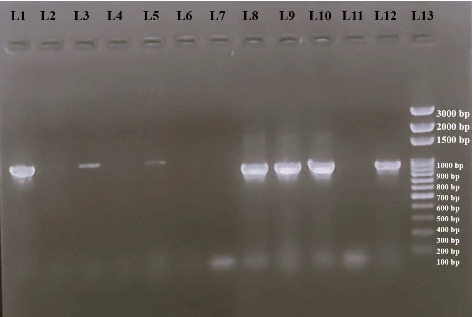
Visualization of PCR products of *bla_CTX_* and *bla_TEM-52_* genes of *E. coli* isolates. **L1 to L6:** PCR products of *bla_TEM-52_* gene of *E. coli* isolates. **L7 to L12:** PCR products of *bla_CTX_* gene of *E. coli* isolates. **L13:** 100-bp DNA ladder. **L1 (FZ100), L3 (L17B), and L5 (L12B):** presence of *bla_TEM-52_* gene in these isolates. **L8 (L8C), L9 (L17B), L10 (L15B), and L12 (L11B):** presence of *bla_CTX_* gene in these isolates.

Following phenotypic determination of the resistance pattern, isolates showing positive results in phenotypic analyses were tested for the presence of antimicrobial resistance genes (ARGs). The seven isolates testing positive for ESBL production were tested for the presence of *bla_CTX_* and *bla_TEM-52_* genes, while two isolates with a positive result in the colistin-resistant test were tested for the detection of *mcr-1* and *mcr-2* genes. All seven ESBL producers were found to harbor at least one of the ESBL encoding genes. Two isolates—L4B and L17B—were confirmed to possess both *bla_CTX_* and *bla_TEM-52_* genes, while two isolates—L11B and L8C—possessed only the *bla_CTX_* gene, and two isolates L12B and FZ100 possessed only the *bla_TEM-52_* gene. The prevalence of resistance-encoding genes is shown in Tables 5 and 6, and Figure 8 illustrates the result of PCR amplification of resistance-encoding genes under UV.

## 4. Discussion

Meat and meat products are widely consumed all over the world as a major source of protein and other vital nutrients. Meat and meat products can be a source of antimicrobial-resistant bacteria and serve as a potential reservoir of antimicrobial-resistant genes, which can be transmitted across larger bacterial populations. The findings of the present study revealed the occurrence of antimicrobial-resistant *E. coli* in meat and meat samples. A total of 120 meat samples were processed, in which *E. coli* was recovered from almost half. Among the two categories of meat samples—raw meat and processed meat products, the recovery of *E. coli* was twice as high in raw meat samples (*E. coli* was recovered from 70% of the raw meat samples, while 28.3% were recovered from processed meat products (frozen meat samples); 42.6% of the *E. coli* isolates recovered were MDR.

WOAH Report 2021 on Antimicrobial Use in Animals revealed that tetracyclines were the predominant antimicrobial consumed for veterinary use worldwide, with 35.3% of the consumption in 2021 [24]. In Nepal, tetracycline accounts for 28% of antimicrobials used in animal husbandry, followed by aminoglycosides at 23% and fluoroquinolones at 15% [5]. Among the total *E. coli* isolates recovered from the meat samples, 57.4% were resistant to tetracycline. Thus, the observed resistance of 57.4% against tetracycline correlates with consumption in veterinary use in Nepal. As per the report by the Veterinary Standard and Drug Regulations Laboratory, Nepal on the consumption trend of antimicrobials as an active ingredient in animal use, there was an increase in consumption of tetracycline between 2018 and 2020 [25]. Tetracycline, ampicillin, and ciprofloxacin represent the three major antimicrobial agents against which 30%–60% of the isolates recovered showed resistance. In the context of Nepal, the use of these three antimicrobial classes is on the rise in animal husbandry. Thus, the observed resistance profile can be interlinked with the overuse and misuse. Based on the result, misuse and overuse of antimicrobial agents in livestock farming can also be one of the contributing factors for the development of resistance in bacteria. A study by Gompo et al. [26] shows that the antimicrobial residue in Chicken carcasses was higher for gentamicin, tetracycline, and fluoroquinolones, and residues of gentamicin, streptomycin, and sulfonamides were detected in milk samples from cows and buffalo. This data verifies that the use of antimicrobials is in both the poultry and livestock sectors. Compared to buffalo and cows, the use of antimicrobials is more prevalent in the poultry sector owing to the short production cycle of broilers. The broiler production cycle lasts between 45 and 50 days; thus, they require specialized care to provide the economic benefit. To prevent the loss, the use of antimicrobials is abundant in poultry.

In the study by Aworh et al. [27], 115 *E. coli* isolates were isolated from 429 samples collected from humans, chickens, and poultry environments/farms. Among these, 47 isolates were obtained from poultry works, 27 from poultry–farm environments, and 36 isolates directly from chickens, with 91.8% of the isolates being MDR. Then, 102 isolates showed the highest resistance against tetracycline, followed by 84.5% resistance against trimethoprim-sulfamethoxazole, 79.1% resistance against streptomycin, and 80% against ampicillin. *mcr-1.1* was detected in two isolates obtained from environmental samples, which had plasmid-mediated colistin-resistance genes [27]. This study underscores the emergence of resistance to antimicrobials as a result of their use in poultry industries.

The AST pattern of *E. coli* isolates revealed that 23 isolates out of 54 (43%) were MDR strains. The observed result depicts a dire situation regarding antimicrobial resistance in the food supply chain.

There can be two possibilities for the development of MDR:

1. The use and overuse of antimicrobials in poultry contributed to the development of MDR. These MDR bacteria, due to unhygienic butchering practices, made their way to raw meat through cross-contamination from the gut and other slaughtering equipment.
2. Contamination comes through human sources during the processing of meat and the preparation of meat products.

As mentioned, the maximum resistance of the isolates was towards tetracycline, followed by ampicillin, ciprofloxacin, and trimethoprim-sulfamethoxazole. The majority of the isolates showing resistance to these antimicrobials were obtained from chicken meat samples. Because the resistance pattern of *E. coli* isolates recovered from raw chicken meat and frozen chicken meat samples is the same, we can consider that the origin of MDR *E. coli* in frozen meat samples can be traced to raw chicken meat used in the preparation of such products. On the contrary, the resistance pattern of uropathogenic *E. coli* from human sources depicts higher resistance to amoxicillin (95.96%) and ampicillin (85.71%) [28]. As per the community-based surveillance carried out to assess resistance patterns in *E. coli* among apparently healthy adults, the resistance pattern revealed higher resistance to ampicillin (40.0%), tetracycline (20.7%), and cefotaxime (15.5%) [29]. The resistance pattern in *E. coli* from human sources is different than those recovered from animal source. Thus, the lack of proper butchering facilities and poor hygiene conditions in the butchering center prevalent in the valley are responsible for the cross-contamination of meat with MDR bacteria and other pathogens from the gut, skin, and waste of the butchered animal.

*β*-Lactams, carbapenems, and colistin represent critically important antibiotics (CIA) that serve as a last line of defense for the treatment of extreme antimicrobial-resistant cases. Resistance to these CIAs signifies an alarming situation, as various clinical cases have been reported. Resistance to these in food-producing animals and their meat implies the use of the CIAs in veterinary practices or cross-contamination through water or humans. Detection of ESBL production revealed the occurrence of seven presumptive ESBL-producing *E. coli* isolates. Five isolates were obtained from raw buffalo meat, while one each from raw chicken meat and frozen meat products. Genomic analysis on seven ESBL-producing isolates for the detection of two ESBL-production genes—*bla_CTX_* and *bla_TEM-52_*—showed that isolates with positive phenotypic expression of ESBL production possessed at least one of the resistant genes. Two isolates—L4B and L17B—were found to possess both *bla_CTX_* and *bla_TEM-52_* genes, while the *bla_CTX_* gene was detected in isolates L11B, L15B, and L8C, and the *bla_TEM-52_* gene was detected in L12B and FZ100 isolates. Phenotypic expression of ESBL production was found to correspond to the presence of at least one resistant gene in bacterial isolates. The prevalence of ESBL-producing *E. coli* in meat samples was found to be 12%. CTX-M, TEM, and SHV represent the common ESBL encoding genes prevalent worldwide. A study by Palmeira and Ferreira [30] revealed ESBL-producing Enterobacteriaceae to be a major threat in cattle around the world. This review article stated that CTM, SHV, and TEM are the most prevalent resistant genes attributed to ESBL in the world. CTX-M is the predominant resistant gene with a number of variations with respect to different origins in different areas around the world [30].

*E. coli* isolates were tested for the phenotypic carbapenem resistance test, in which all isolates were found sensitive to the meropenem and imipenem antimicrobials, which belong to the carbapenem group. The absence of carbapenem-resistant *E. coli* isolates recovered from meat samples highlights a positive aspect of the meat production system. This finding suggests that carbapenem has not been used for veterinary practices as either preventive or curative measures.

Two isolates—L9C and FZ102—were assessed as colistin-resistant *E. coli* among all isolates from meat samples. Following phenotypic expression of resistance against colistin, molecular analysis was performed for the determination of the presence of colistin-resistant encoding genes in the isolates. The major concern regarding antimicrobial resistance is horizontal gene transfer (HGT). ESBL, carbapenemase, and colistin resistance-encoding genes are predominantly present in plasmids. As plasmids can be transferred between bacterial populations, the presence of these genes can be a major concern. Resistance acquired by one strain of bacteria is not limited to itself. Under favorable conditions, bacteria can transfer the resistant gene among a wide range of recipient bacterial strains, thus causing the dissemination of the ARGs.

These 2 isolates—L9C and FZ102—were analyzed for the presence of colistin-resistant genes—*mcr-1* and *mcr-2*. Polymerase chain reaction was carried out targeting the specific genes, *mcr-1* and *mcr-2*, and visualized by agarose gel electrophoresis for confirmation of target DNA. PCR analysis revealed that both isolates possessed the *mcr-1* gene, while the *mcr-2* gene was not detected in the isolates.

According to the European Union Summary Report on Antimicrobial Resistance in zoonotic and indicator bacteria from humans, animals, and food in 2020/2021, the prevalence rate of third-generation cephalosporins was relatively low. In countries that reported presumptive ESBL and/or AmpC-producing *E. coli* isolates, the prevalence rate ranged around 0.6%–8.7% in pigs, 0.07%–3.5% in isolates from calves, 0.6%–6.3% in turkeys, and 0.6%–7.1% in broilers. In the period 2020–2021, the prevalence rate of colistin resistance in *E. coli* for all animals was very low, 1.1% in 143 isolates out of 13,239 isolates in the EU region [31]. Similarly, the European Union Summary Report on Antimicrobial Resistance in zoonotic and indicator bacteria from humans, animals, and food 2022/2023 revealed *E. coli* isolates recovered from four investigated animals and their derived foods were high to very high median level resistant to ampicillin, sulfamethoxazole, trimethoprim, and tetracycline. *E. coli* isolates were low to moderate level resistant to chloramphenicol. MDR assessment in the indicator *E. coli* isolates showed that 40% of the isolates recovered from the broiler sample were MDR. In European member states, the prevalence of a variety of ESBL and AmpC encoding genes was recorded in 2022 and 2023. *bla_CTX-M-1_* and *bla_CTX-M-15_* were the prevalent ESBL encoding genes overall. *bla_CTX-M-1_* and *bla_SHV-12_* were the most common ESBL encoding genes detected in broilers, and a reverse trend was seen in meat from broilers. In the context of pigs, cattle, and bovines, *bla_CTX-M-1_* and *bla_CTX-M-15_* were the common variants of ESBL genes detected [32].

Overall, Nepal is in a vulnerable state in the context of AMR in food-producing animals. *bla_CTX_* was found to be a prevalent ESBL-encoding gene in *E. coli* isolates recovered, and the detection of *mcr* genes raises concern over the effectiveness of the colistin ban.

*E. coli,* being resident bacteria of the animal and human gut lining, the presence of MDR strains and resistance encoding genes in them indicates the probability of other microorganisms acquiring resistance determinants. *S. aureus* and *Salmonella* spp. are common pathogens of zoonotic origin that can infect humans through the consumption of contaminated foods of animal origin. The probability of development of antimicrobial resistance in these pathogens is high with regard to the prevalence of MDR and resistance genes in *E. coli* from animal origin. Humans, animals, and the environment are interrelated; thus, AMR must be tackled within the framework of the “One Health Approach” [33]. Dissemination of AMR can occur from animals to the environment to humans or directly from animals to humans through food. Use of antimicrobials in veterinary practice must be justified, and misuse and overuse must be restricted, as the threat of AMR is imminent. Cephalosporins, carbapenems, and colistin are critically important antimicrobials, and their use must be justified as new drug discovery is difficult. A proper action plan and proper dissemination of knowledge regarding AMR are crucial in tackling the threat posed by them.

## 5. Conclusions

Moderate-level prevalence of MDR *E. coli* (42.6% out of 54 isolates), along with detection of resistance determinants for ESBL (12.9%) and colistin (3.7%), raises a serious concern. These isolates can act as a potential reservoir for these resistance determinants and can play a role in the dissemination of the markers across other bacterial populations. Thus, it is essential to monitor the use of antimicrobials in veterinary use by actively controlling the trade of these antimicrobials by regulatory bodies, along with awareness development among the farmers, processing industries, and retail owners along the meat value chain. A scientific study on the overall meat value chain can be crucial in depicting the real scenario of AMR in meat and its associated threat to humans.

## Figures and Tables

**Table 1:** PCR conditions for amplification of a 584-bp fragment of the 16S rRNA gene of *E. coli* [20].

**PCR steps**	**Time–temperature conditions**		**Reference**
Initial denaturation	95°C for 5 min		[20]
Denaturation	94°C for 1 min	35 PCR cycles	
Annealing	55°C for 90 sec		
Extension	72°C for 1 min		
Final extension	72°C for 10 min		

**Table 2:** Reaction mixture prepared for PCR of target genes.

**Component**	**Volume**	**Final concentration**	**Reference**
FIREPol Master Mix Ready to Load (5x)	4 *μ*L	1x	[22]
Forward primer (10 *μ*M)	1 *μ*L	0.5 *μ*M	
Reverse primer (10 *μ*M)	1 *μ*L	0.5 *μ*M	
DNA template	1 *μ*L		
Nuclease-free water (NFW)	13 *μ*L		
Final PCR volume	20 *μ*L		

**Table 3:** PCR conditions for amplification of antimicrobial-resistant genes (ARGs) in *E. coli* isolates.

**PCR steps**	**Time–temperature conditions**			**References**
	**Set A: mcr-1 and 2**	**Set B: ESBL genes**	**Set C: carbapenemase**	
Initial denaturation	94°C for 3 min	94°C for 4 min	94°C for 3 min	[23]
Denaturation	94°C for 45 s	94°C for 1 min	94°C for 45 s	
Annealing	60°C for 1 min	54 °C for 1 min	56°C for 1 min.	
Extension	72°C for 3 min	72°C for 2 min	72°C for 1 min	
PCR cycles	30 cycles	30 cycles	36 cycles	
Final extension	72°C for 5 min	72°C for 2 min	72°C for 5 min	

**Table 4:** Lists of primers and their sequence, along with target genes in test organisms, and the size of PCR products.

**Primers**	**Primer sequence**	**Product size (bp)**	***Target gene***	***References***
MCR1-F2	AGTCCGTTTGTTCTTGTGGC	320	*mcr-1*	[23]
MCR1-R2	AGATCCTTGGTCTCGGCTTG			
MCR2-F	CAAGTGTGTTGGTCGCAGTT	715	*mcr-2*	
MCR2-R	TCTAGCCCGACAAGCATACC			
blaTEM-52 F	ATAAAATTCTTGAAGACGAAA	1080	*bla_TEM-52_*	
blaTEM-52 R	GACAGTTACCAATGCTTAATC			
blaCTX-F	CCCATGGTTAAAAAACACTGC	950	*bla_CTX_*	
blaCTX-R	CAGCGCTTTTGCCGTCTAAG			
IMP-F	GGAATAGAGTGGCTTAAYTCTC	232	*bla_IMP_*	
IMP-R	GGTTTAAYAAAACAACCACC			
OXA-F	GCGTGGTTAAGGATGAACAC	438	*bla_OXA-48_*	
OXA-R	CATCAAGTTCAACCCAACCG			

**Table 5:** Distribution of ESBL genes—*bla_CTX_* and *bla_TEM-52_* in *E. coli* isolates.

**S.N.**	**Isolates**	**ESBL-producing genes**	
		***bla***_***CTX***_	***bla***_***TEM-52***_
1	L4B	Present	Present
2	L11B	Present	Absent
3	L12B	Absent	Present
4	L15B	Present	Absent
5	L17B	Present	Present
6	L8C	Present	Absent
7	FZ100	Absent	Present

**Table 6:** Distribution of colistin-resistant genes—*mcr-1* and *mcr-2* in *E. coli* isolates.

**S.N.**	**Isolates**	**Colistin-resistant genes**	
		***mcr-1***	***mcr-2***
1	L9C	Present	Absent
2	FZ102	Present	Absent

## Data Availability

The data that support the findings of this study are available from the corresponding author upon reasonable request.

## Nomenclature

AMR: Antimicrobial Resistance
ATCC: American Type Cell Culture
CLSI: Clinical and Laboratory Standards Institute
ESBL: Extended Spectrum Beta-Lactamase
DFTQC: Department of Food Technology and Quality Control
CHDS: Center for Health and Disease Studies
MDR: Multidrug Resistance
WHO: World Health Organization
EDTA: Ethylene Diamine Tetra-acetic Acid
TE: Tris-EDTA
CTAB: Cetyltrimethylammonium Bromide
PCR: Polymerase Chain Reaction
WOAH: World Organization for Animal Health
CIAs: Critically Important Antimicrobials
HGT: Horizontal Gene Transfer
